# Advances in single‐cell sequencing and its application to musculoskeletal system research

**DOI:** 10.1111/cpr.13161

**Published:** 2021-12-09

**Authors:** Yongxiang Zhang, Jingkai Wang, Chao Yu, Kaishun Xia, Biao Yang, Yuang Zhang, Liwei Ying, Chenggui Wang, Xianpeng Huang, Qixin Chen, Li Shen, Fangcai Li, Chengzhen Liang

**Affiliations:** ^1^ Department of Orthopedics Surgery The Second Affiliated Hospital Zhejiang University School of Medicine Hangzhou Zhejiang China; ^2^ Zhejiang Key Laboratory of Bone and Joint Precision and Department of Orthopedics Research Institute of Zhejiang University Hangzhou Zhejiang China; ^3^ The MOE Key Laboratory of Biosystems Homeostasis & Protection and Zhejiang Provincial Key Laboratory for Cancer Molecular Cell Biology Life Sciences Institute Zhejiang University Hangzhou China; ^4^ Hangzhou Innovation Center Zhejiang University Hangzhou China

**Keywords:** cell heterogeneity, musculoskeletal system, single‐cell multi‐omics, single‐cell sequencing

## Abstract

In recent years, single‐cell sequencing (SCS) technologies have continued to advance with improved operating procedures and reduced cost, leading to increasing practical adoption among researchers. These emerging technologies have superior abilities to analyse cell heterogeneity at a single‐cell level, which have elevated multi‐omics research to a higher level. In some fields of research, application of SCS has enabled many valuable discoveries, and musculoskeletal system offers typical examples. This article reviews some major scientific issues and recent advances in musculoskeletal system. In addition, combined with SCS technologies, the research of cell or tissue heterogeneity in limb development and various musculoskeletal system clinical diseases also provides new possibilities for treatment strategies. Finally, this article discusses the challenges and future development potential of SCS and recommends the direction of future applications of SCS to musculoskeletal medicine.

## Graphical abstract

Single cell sequencing (SCS) technologies have superior abilities to analyze cell heterogeneity at a single‐cell level, which have elevated omics research to a higher level. Meanwhile, the applications of SCS have enabled many valuable discoveries, and musculoskeletal system offers typical examples. Single cell sequencing of bone, muscle, articulation and cartilage produces a great deal of valuable data. Through integration analysis and visualization, the musculoskeletal system heterogeneity is revealed, which provide new clues for regeneration and disease treatment.

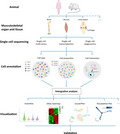

## 1 INTRODUCTION

The basic unit of an organism is cell, and multicellular lives in nature begin with a single cell. Although preliminary estimates suggest that every person is composed of at least 37.2 trillion cells,^1^ a deeper understanding is still very limited concerning the functions of those cells. Thus, the Human Genome Project (HGP) was officially launched in 1990 to unravel the genetic code of all of the approximately 25,000 genes in the human body and map the human genome atlas.^2^ In 2001, the publication of a working draft of the human genome was considered a milestone in HGP.^3^ The Human Cell Atlas (HCA) was launched in 2007 to further describe all human cells.^4^ HCA integrated the information of cell types, number, location, relationship and molecular composition that facilitates to describe the cellular basis of health and disease. Gene sequencing is considered as a highly reliable method for analysing cell genetic attribution.^5^ The earliest method of gene sequencing can be traced back to in 1977,^6^ where ‘Sanger sequencing’ was a revolutionary technology, which ushered the era of gene sequencing research. Over the last several decades, due to its accuracy, ‘Sanger sequencing’ has been widely incorporated to scientific investigations such as HGP and diagnosis of clinical genetic diseases.^7^ However, because of its complexity and high cost, investigators have been trying to develop more efficient sequencing methods.

Next‐generation sequencing (NGS) is the second revolutionary innovation of traditional gene sequencing.^8^ NGS has the characteristics of high throughput and low cost of per base, also known as massively parallel sequencing (MPS).^9^ Further advances in computer science and technology have enabled development of third‐generation gene sequencing, with the ability of high‐throughput, single‐molecule sequencing. It can produce genome assemblies of unprecedented quality.^7^ In 2009, Tang first reported a method for investigating the mRNA transcriptome using high‐throughput sequencing in a single cell. This achievement is considered the beginning of widespread application of SCS technologies in scientific research.^10^ Thanks to the application of SCS, cell biology and molecular biology had made great discovery in recent years. In 2018, researchers used SCS to create dynamic maps of gene expression during early embryonic development of zebrafish and frogs. Through integrating data on time scales in minutes to hours, describing the cells one by one, and tracking the eventual formation of embryo, investigators were able to build a complete map that revealed the entire developmental process from a single cell to an entire organism.^11^, ^12^ SCS can deepen our understanding of various aspects of cell function, such as tumorigenesis,^13^ nerve degeneration,^14^ immunology,^15^ cell differentiation^16^ and gene expression.^17^

Limb development is a fundamental event in musculoskeletal system. Researches reveal that mesenchymal stem cells (MSCs) can act as bone progenitor cells in bone marrow. Skeleton development begin with the migration of mesenchymal cells derived from the embryonic lineage to the sites of future bone.^18^ Further, researches reveal that transcription factor SRY (sex determining region Y)‐box 9 (Sox9) plays a critical role in inducing osteogenic differentiation of MSCs.^19^, ^20^, ^21^ Moreover, many morphogenetic or growth factors, such as WNTs, Hedgehogs, Notch, VEGF, FGFs, IGF‐1, TGF‐β and PTHRP, have been found to be involved in the regulation of endochondral bone formation.^22^ For musculoskeletal disease research, trauma, pain and limb malformation are main issues. More specifically, bone fracture, joint injury, osteoarthritis and intervertebral disc degeneration are the common clinical diseases. The analysis of cell heterogeneity and activation of specific types of stem cells have become hot issues in this field.^23^ Stem cells are found in the bone marrow and periosteum. The stem cell population is made up of heterogeneous cells. Previous studies have focused on histomorphology and gene expression regulation, which skipped the cellular level. Some important information is hidden in heterogeneous cell populations. By combining SCS and lineage tracing, a unique subpopulation of periosteum stem cell is identified to contribute to bone regeneration.^24^ More importantly, the contribution of periosteal stem cells to bone regeneration is higher than that of bone marrow stem cells.

In this review, we first outline the major scientific issues and recent advances on the musculoskeletal system. Second, we discuss single‐cell technologies in detail, including its technical features, superiority and its application in multi‐omics studies. From our perspectives, cell heterogeneity has become the focus in limb development and clinical diseases of musculoskeletal system, and the application of SCS will provide us with unprecedented understanding of these issues. Moreover, the challenges and possible future directions of single‐cell technologies are also discussed.

## 2 THE MUSCULOSKELETAL SYSTEM

### 2.1 Overview

**FIGURE 1:**
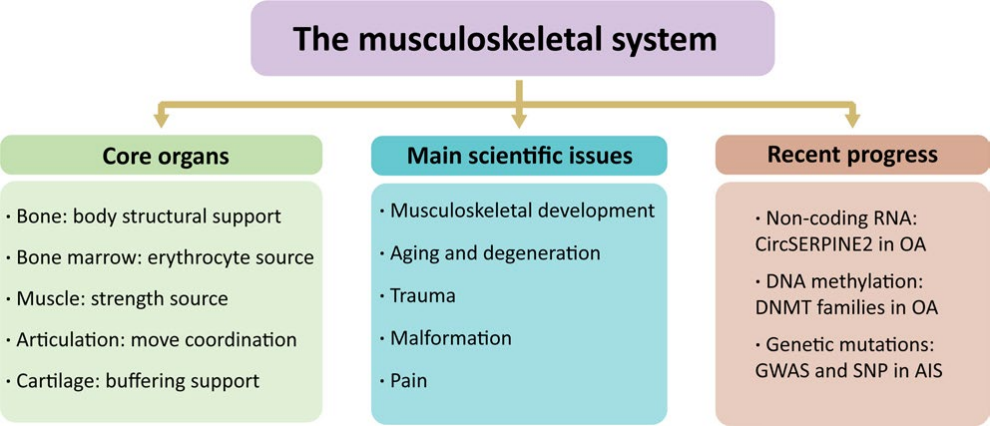
An overview of the research issues of the musculoskeletal system

Musculoskeletal system consists of bone, muscle, articulation, cartilage and other connective tissue that stabilize or connect bones (Figure 1).^25^ In addition to supporting the body's weight, bone and muscle work together to keep the body in position or to produce controlled and precise movements.^26^

Bones provide structural support for the entire body and also protect the internal organs. Red bone marrow in long or flat bones lacunae produces blood cells. There are many attachments of muscle to the bones, which work as levers to change the magnitude and direction of the strength produced by the muscles.^27^ Articulations play an important role in movement coordination. The stability and range of different articulations movement in the human body vary greatly, depending on the capacity of the associated muscles, tendons and ligaments. Cartilage is a kind of supportive buffering connective tissue.^28^ Depending on its intercellular attribution, cartilage can be divided into three types: hyaline cartilage, elastic cartilage and fibrous cartilage. These three types of cartilage have different properties and functions in different parts of the body.

### 2.2 Main scientific issues

The study of the musculoskeletal system is mainly divided into musculoskeletal development and clinical diseases. Chronic pain is the most common symptoms in orthopaedic clinics. In addition to injury‐induced pain, degeneration and ageing of bones, cartilage and intervertebral discs (IVDs) are the main causes of pain. In the general population, bone ageing results in loss of volume and mass, usually manifested as osteoporosis and an increased risk of fracture.^29^, ^30^ Cartilage degeneration is common in joints. Degeneration of chondrocytes in the knee joint is a typical example. Ageing decreases cartilage thickness, which will lead to osteoarthritis (OA). Loss of IVD structural integrity can result in loss of IVD height, leading to collapse and compression of the spine and clinical symptoms, such as lower back pain.

Bone marrow MSCs can be stimulated to form osteoblasts, myocytes and fibroblasts. Change in the number or activity of these cells will affect a range of musculoskeletal tissues. Older MSCs exhibit a state of irreversible growth stagnation or senescence. Expression of p53, p21 and ageing‐related β‐galactosidase was increased compared to that in young MSCs, perhaps due to the down‐regulation of age‐related osteogenic genes, including Runx2 and osteocalcin.^29^ Age‐related changes in sex hormones can also affect normal bone biology. For example, postmenopausal women have gradually decreased oestrogen, while osteoclasts being released in response to oestrogen inhibition, increasing overall bone resorption.^31^ During ageing, the molecular structure of articular cartilage changes in the same time. The most significant changes are reflected in decreased proteoglycans, aggrecan and water content of chondroitin sulphate (CS), crushed core protein well as increased keratan sulphate concentrations. Similarly, ageing IVDs show increased expression of matrix metalloproteinases (including MMP3 and MMP7) and disintegrin. These enzymes degrade collagen and prostaglandins, leading to the dehydration and disintegration of the extracellular matrix (ECM). Under these conditions, the ECM becomes granular, cracked or torn. The structural strength of the annulus fibrosus (AF) decrease makes it easy for the nucleus pulposus (NP) to excrete.^32^, ^33^

In addition, malformation is a common concern of musculoskeletal system. The representative disease is scoliosis, among which adolescent idiopathic scoliosis (AIS) is the most common clinical type, occurring in 0.5%–3.0% of children.^34^ It is currently believed that AIS is a multi‐factor disease with genetic predisposition.^35^ However, the chromatin‐level pathogenesis of scoliosis remains uncertain.

### 2.3 Recent progress

High‐throughput sequencing technologies have been widely used in musculoskeletal system. These applications have led to many discoveries in the regulation of gene diagnosis and non‐coding genes in disease. For example, a variety of factors involve and regulate the process of OA. Recently, a novel CircRNA (CircSERPINE2) is identified in an RNA‐seq comparing total RNA in several groups of clinical OA with that of normal control tissues. Transfection experiments suggest that CircSERPINE2 could reduce chondrocyte apoptosis and promote ECM synthesis. Further studies found that CircSERPINE2 plays a role in OA by targeting miR‐1271 expression. The presumed target gene of miR‐1271 is ERG [E26 transference specific (ETS)‐related gene]. The CircSERPINE2‐miR‐1271‐ERG axis is recognized as a new target for OA prevention and treatment.^36^ Likewise, OA is a complete joint disease, and treatment strategies need to be able to target a wide range of relevant cell signalling pathways in cartilage synovium and bone. Studies over the past decade have revealed a large number of high‐risk genetic loci for OA, which are predicted to increase disease risk by modulating the expression of target genes. Many risk loci are associated with epigenetic mediators.^37^ Specifically, epigenetic factors can regulate gene expression by influencing chromatin status, and the regulation is accompanied by changes of multiple signal transduction pathways. For example, DNA methylation is catalysed by DNA methyltransferase families (DNMT1, DNMT3A and DNMT3B). A study found DNMT3A expression was up‐regulated in a subset of OA patients (*n* = 71) compared with the normal control group (*n* = 32), and DNMT3A knockout was observed to reduce the catabolism of interleukin‐1β (IL1‐β) in the ECM.^38^ However, DNMT3B had the opposite effect. It inhibits 4‐aminobutyrate amino transferase (ABAT) degradation, while increased ABAT expression leads to enhance catabolic activity. Enzymes that undergo epigenetic changes can have a wide range of simultaneous effects in OA.^39^ These effects cannot be achieved by targeting a single pathway.

In recent years, researchers have attempted to study the genetics of spinal malformations to explore their pathogenesis. Wu et al used comparative genomic hybridization to analyse congenital scoliosis in Han populations for the first time.^40^ These investigators found large DNA deletions in the 16P11.2 region of the genome of patients with sporadic congenital scoliosis, and sequencing analysis confirmed that TBX6 gene in this deletion region was the source of this pathology. Moreover, the rare combination of null mutations and subtype alleles in TBX6 accounts for 11% of cases of congenital scoliosis in Han populations. This finding provides a theoretical basis for early diagnosis and genetic counselling of high‐risk population. Moreover, Zhu et al used genome‐wide association analysis (GWAS) to locate loci were 1p36.32, 2q36.1, 18q21.33 and 10q24.32 associated with AIS in Han Chinese girls.^41^ The discovery that these genes are closely related to bone growth and osteoblastic differentiation provides new insights into the genetic causes of AIS.

## 3 SINGLE‐CELL SEQUENCING TECHNOLOGIES

### 3.1 SCS can analyse cell heterogeneity, even in small samples

SCS technologies have obvious superiorities. Generally, most sequencing requires a sample size of at least one milligram, but the amount of DNA or RNA in a single cell is very small. For example, a typical cancer cell contains 6~12 pg of DNA and 10~50 pg of RNA (1%~5%mRNA).^42^ In the past, genetic material from over ten thousand cells had to be extracted simultaneously to meet the sample size required for sequencing analysis. Yet, random expression of genes coding for proteins and metabolites causes cell heterogeneity, and genetic information in cells with the same phenotype may differ significantly. Simultaneous analysis of multiple cells will equalize cell signals and obscure cell heterogeneity.^43^ In assessing the genetic clonal structure of tumours at the genome‐wide level, the heterogeneity of tumour cells makes batch sequencing difficult and inaccurate. SCS is revolutionary because it can quantify all transcriptomes expressed in a single cell. High‐resolution analysis can reveal intercellular differences that would normally be masked by batch sampling methods, providing a completely unbiased strategy for identifying and characterizing different cell populations. SCS also enables reliable transcriptome analysis at the level of single cell. Some research focuses on limited number of cells, such as unicellular microorganisms or rare cells; in these cases, genetic information is especially difficult to obtain by conventional high‐throughput sequencing. Yet, SCS can make these studies possible.^44^, ^45^

### 3.2 Single‐cell technologies and procedures

**FIGURE 2:**
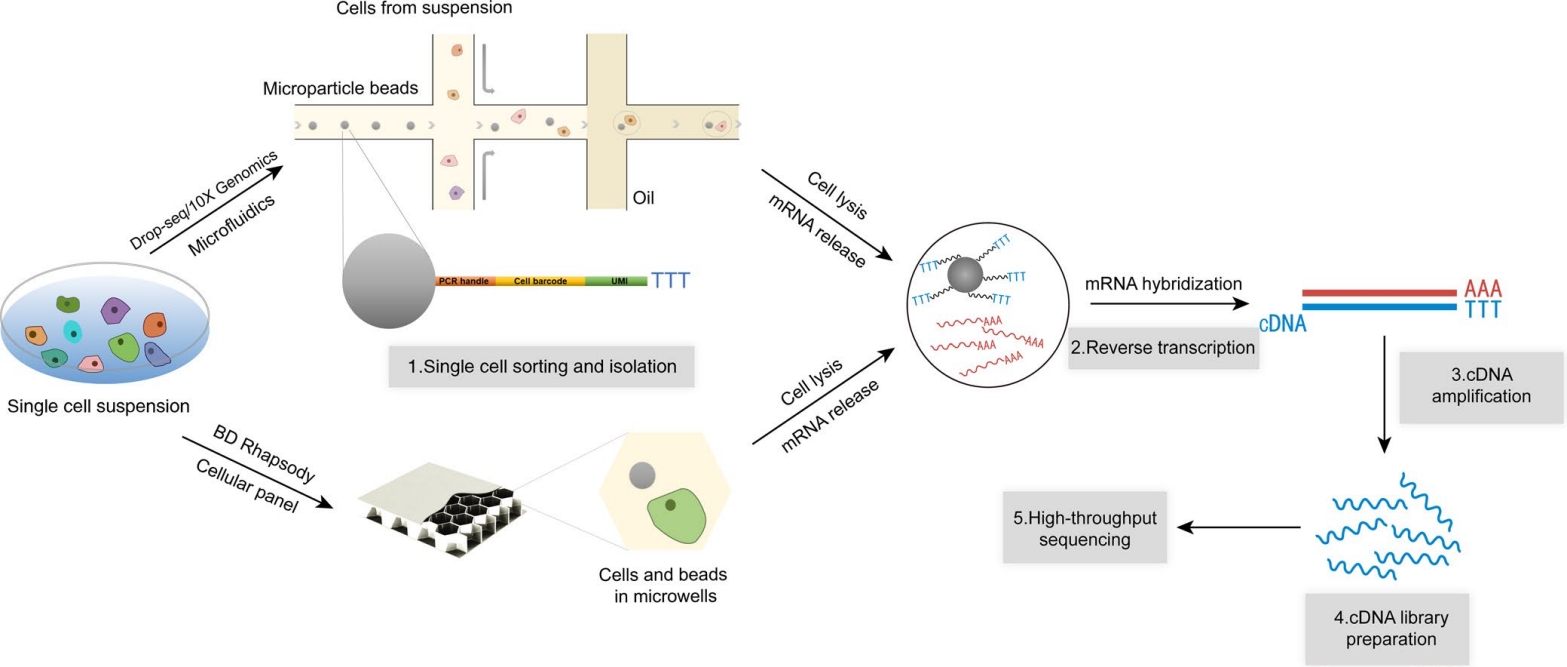
Basic procedures of single‐cell transcriptome sequencing (scRNA‐seq) and three common single‐cell sorting platform. ScRNA‐seq generally involves five basic procedures: single‐cell isolation, reverse transcription, cDNA amplification, library preparation and high‐throughput sequencing. Drop‐seq, 10X Genomics and BD Rhapsody are the most widely used single‐cell sorting platforms. They use microparticle beads with sequences linked by different cell barcodes, UMI and Oligo dT. These beads can capture mRNAs with poly A tails for reverse transcription and cDNA library preparation. Finally, all transcriptome information of a single cell is obtained by high‐throughput sequencing

Single‐cell transcriptome sequencing (scRNA‐seq) is most widely used in the field of SCS technologies, generally including the following five steps: (1) Single‐cell isolation, (2) Reverse transcription, (3) cDNA amplification, (4) Library preparation and (5) High‐throughput sequencing.^46^ A schematic diagram of the general process is shown in Figure 2. Isolation of a single cell is the most critical step in which the target cells must be accurately located and isolated from the samples. The initial two methods for single‐cell separation, continuous dilution and micromanipulation, both had the disadvantages of low throughput and cell susceptibility to mechanical damage. More recent cell separation methods have included magnetic activated cell sorting (MACS),^47^ fluorescence activated cell sorting (FACS)^48^ and microfluidics.^49^ Notably, microfluidics involves fewer step and has the advantages of high analytical sensitivity and specificity as well as high throughput. Drop‐seq is a representative method to capture single cell by microfluidics.^50^ This method uses barcode beads (Figure 2). The oligonucleotides on the beads include handle primer for PCR amplification, cellular barcode UMI (unique molecular identifier) that recognizes all oligonucleotides in a single cell and oligonucleotides (Oligo dT) that capture single‐cell mRNA molecules. Based on Drop‐seq, 10X Genomics was developed as a widely used SCS library preparation platform. 10X Genomics is similar to Drop‐Seq method of capturing single cells, but it is integrated into an oil droplet‐based microfluidic device that effectively captures a larger cell count with higher sensitivity.^51^ In addition, BD Rhapsody is the another widely used SCS platform. Instead of microfluidic channel emits cells and collides with the outgoing magnetic beads, BD Rhapsody uses CytoSeq unique cellular panel.^52^ First, the cell suspension is injected through the injection hole. It naturally subsides into the reaction hole. Then, beads are inserted through the injection hole in the same way to capture the cells in a single reaction hole. This method allows cells to be physically isolated from each other. Furthermore, in 2017, Chen et al. developed a new technology for single‐cell space transcriptome sequencing: Geo‐seq.^53^ This method retains the original spatial information in single cell and elucidates the heterogeneity and spatial variation of cells. This approach has great potential in stem cell and embryonic development research. Recently, Georg Seelig's team developed a method called SPLiT‐seq.^54^ The cell acts as a reaction chamber, immobilizing the cell or nucleus, allowing efficient sample reuse. This is a low‐cost method that does not require special customized instruments and enables the use of single‐cell technology in a greater range of laboratory research.

### 3.3 Single‐cell multi‐omics

The emergence of single‐cell omics has provided unprecedented insight into embryonic development and the development of disease. Genomics, transcriptomics and proteomics are three important aspects of single‐cell omics technologies, which allow us to understand the characteristic of the transmission and expression of genetic information with higher resolution and accuracy. Single‐cell whole‐genome sequencing is an effective method for screening single‐cell SNP (single nucleotide polymorphism) and CNV (copy number variation). It is of great significance for the detection or prediction of tumour‐driven mutations because it can evaluate the overall quality of DNA and annotate the variation.^55^ For instance, hepatocellular carcinoma (HCC) is considered to be a cancer of genetic and phenotypic diversity. Duan et al profiled 96 tumour cells and 15 normal liver cells using single‐cell whole‐genome sequencing.^56^ They found CNV occurred in the early stages of HCC and remained relatively stable during tumour progression. Single‐cell transcriptome sequencing is a technology aiming to study of cell heterogeneity based on bulk transcriptome sequencing.^57^, ^58^ However, if the cell population is analysed only from a single‐omics approach at a time, only local conditions in the gene regulatory network can be detected. The overall landscape cannot be accurately provided. Single‐cell multi‐omics is developed by combining various single‐cell single‐omics technologies. Genetic information, chromatin state, RNA expression and protein abundance can be analysed simultaneously to help us further understand the regulatory mechanism of gene expression.^59^, ^60^

Researchers have developed a variety of single‐cell multi‐omics technologies. Specifically, DNA‐mRNA sequencing (DR‐seq) a sequencing method, lyses a single‐cell and simultaneously amplifies the DNA and RNA.^61^ The suspension is divided into two samples, one for RNA sequencing and the other for genomic DNA sequencing. This method keeps DNA and RNA amplifying together, meanwhile minimizing nucleic acid loss. Genome and transcriptome sequencing (G&T‐seq) is another important method.^62^ In G&T‐seq, mRNA and DNA are physically separated from fully lysed cells using magnetic beads coated with short oligonucleotide sequences that bind mRNA. DNA and mRNA are then amplified and sequenced. This method keeps mRNA and DNA separate, allowing researchers to analyse each molecule using their chosen scheme. However, this approach can lead to loss of nucleic acids. DR‐seq and G&T‐seq are lysing whole cell, while simultaneous isolation of genomic DNA and total RNA (SIDR) was developed as a cell membrane lysis method.^63^ The method captures and labels cells with magnetic beads that bind to specific antibodies. A hypotonic solution is used to destroy the plasma membrane and release total RNA, leaving the nuclear fibrous layer intact and genomic DNA (gDNA) remaining in the nucleus. The lysis suspension is then placed on a magnetic frame, where the supernatant is total RNA and the precipitate was gDNA. They can physically isolate of total RNA in gDNA, including mRNA and non‐coding RNA (especially long non‐coding RNA, LncRNA). In addition, SIDR has a more accurate comparison ratio than DR‐Seq, and this method can be applied to more comprehensive studies of cell heterogeneity and complexity.

Notably, single‐cell multi‐omics has a significant application prospect. First, cell subtypes can be identified from heterogeneous cell populations. Parallel measurements of different omics can provide non‐overlapping information about the identity of the cell, enabling more detailed and accurate classification of the cell population. Second, tracking the cell lineages throughout the development of multicellular animals is one of the most important aspects of developmental biology. Lineages can be traced through mutations or epigenetic modifications of DNA during cell division.^64^ Matching single‐cell transcriptome data can also reveal the changes in gene expression and the fate of cells during proliferation and differentiation.

### 3.4 Single‐cell ATAC‐seq and epigenetics

Nucleosomes are the basic structural units of chromatin in eukaryotes. DNA binds to histones to form nucleosomes, which are further folded and compressed to form chromatin. Both replication and transcription of DNA require the opening of tightly packed chromatin structures to allow regulatory molecules to bind to DNA. The opened section of the chromatin is called accessible chromatin. The search for accessible chromatin regions is the most critical step in the study of transcriptional regulation. William et al used DNA transposase in combination with high‐throughput sequencing to study chromatin accessibility, an approach known as ATAC‐seq (Assay for Transposase Accessible Chromatin with high‐throughput sequencing).^65^ ATAC‐seq is a genome‐wide measure of chromatin accessibility, which can obtain genome‐wide protein binding site information. ATAC‐seq can be used to screen for unknown specific transcription factors.

Single‐cell ATAC‐seq (scATAC‐seq) is a single‐cell resolution method for the study of chromatin accessibility. Specifically, sequences in open chromatin regions contain a large amount of motifs information about transcriptional initiation. Motif information can be found in the database for corresponding regulatory transcription factors, thus providing information for downstream experimental design. Frisén et al selected populations of resident non‐nerve cells in the mice spinal cord for scRNA‐seq and scATAC‐seq.^66^ They observed that the motifs of typical oligodendrocyte lineage transcription factors OLIG2 and SOX10 were highly available not only in oligodendrocyte progenitor cells (OPC) but also in ependymal cell populations.^67^ However, OLIG2 and SOX10 are expressed in oligodendrocytes but not in ependymal cells. Their subsequent experiments confirmed that OLIG2 expression in ependymal cells activates a potential oligodendrocyte lineage program after spine cord injury (SCI). ScATAC‐seq revealed that specific transcription factor OLIG2 was the core basis of Frisén's study. Because ATAC‐seq is more repeatable and easier to operate than traditional methods, it has become the preferred method to study chromatin accessibility.

Environmental conditions affect the metabolism of cells and organisms. With changing conditions, the organism tries to adjust its metabolism in response to the environment. Gene mutations may also change biological differentiation pathways.^68^ Epigenetic research explores the complex interaction between environmental conditions and genetic factors to illuminate the pathogenesis of disease.^69^ Extensive evidence supports the view that epigenetic mechanisms (DNA methylation, post‐translational modifications of histone tails and non‐coding RNAs) are involved in the differentiation of bone cells.^70^ Currently, epigenetic studies are mainly conducted at the bulk level. Different cell types with distinct epigenetic characteristics are often mixed together in tissues or organs, thus presenting a challenge for research.

NGS has greatly facilitated the study of epigenetics by enabling the study of DNA methylation at single‐base resolution,^71^ thus elucidating methylation patterns in the entire genome.^72^ Wang et al developed CoBatch,^73^ a single‐cell ChIP‐Seq technology that is universal, easy to operate and of high quality. The method used the fusion Protein PAT (Protein A‐Tn5) to recognize and cut specific genomic regions to which antibodies bind, and combined with the barcode label to achieve high‐throughput single‐cell capture. Single‐cell techniques can reveal the epigenetic heterogeneity of cells and describe the DNA methylation characteristic of cell subsets.^74^ In one study, Angermueller et al observed methylation heterogeneity in two enhancers of mouse ESCs, Esrrb1, and found a negative correlation between methylation level and gene expression.^75^ As mentioned above, of SCS technologies have a superior ability to analyse rare cells. One research focused targeted cells that develop in early mammalian embryos, including especially early human embryonic cells. The epigenome of these cells changes rapidly and significantly during development after fertilization.^76^, ^77^ Therefore, it is important to elucidate epigenetic status by using epigenomic mapping techniques in single‐cell level.

## 4 CELL HETEROGENEITY IN THE MUSCULOSKELETAL SYSTEM

### 4.1 Limb development

Bone and muscle are the most principal organs of musculoskeletal system. In the past, researches on skeleton biology had met with several obstacles. The chemical methods used for treatment of highly cellular tissues such as brain, liver and kidney, may not be applicable to skeleton tissue,^78^ because some solutions cannot penetrate the extracellular matrix to reach bone cells, especially in mineralized tissue.^79^ This problem is recognized in skeleton biology research, and a variety of tissue‐specific RNA separation methods have been developed for bone,^80^, ^81^ cartilage,^82^ ligament and tendon.^83^ Exploring the potential stem cell properties of the periosteum, Debnath et al found that a number of groups of mesenchymal cells are identified with expressed Ctsk (cathepsin K) in the periosteum of long bones.^84^ ScRNA‐seq analysis was performed to divide the cell pool into four groups, one of which expressed progenitor cell/stem cell markers such as Sox9 and Col2a1. Their findings reveal a type of periosteum stem cell (PSC) exists in long bones and skulls of mice. PSC shows clone pluripotency and self‐renewal abilities, and it locates at the top of the differentiation hierarchy. Further research found that the human periosteum also contains a cell population similar to PSC. Moreover, muscle stem cell (MuSC) is required for skeletal muscle development, growth and regeneration.^85^ Stefania et al identified the discrete transcriptional programs of homeostatic MuSC, injured MuSC and primary myoblasts (PMs) by scRNA‐seq. A pseudotime dynamic map from MuSC initiation to PMs was delineated.^86^ Zi et al used scRNA‐seq to study the dynamic transcriptional characteristic of cells at different time points (E10.5, E12.5 and E15.5) during the process from limb bud initiation to the basic formation of musculoskeletal system tissues in mouse.^87^ Pseudotime analysis showed that SCX^+^ HOXD13^+^ musculoskeletal stem progenitor clusters exists in the early stage of limb development. These clusters form distinct branching pathways corresponding to hard and soft connective tissue differentiation during late development. Similarly, Chan et al combined scRNA‐seq data of mouse skeletal stem cell (mSSC) and human skeletal stem cells (hSSC) to identify hSSC characteristic, meanwhile revealed species conservation in skeletogenesis.^88^ Furthermore, He et al discovered a self‐renew cell cluster, embryonic skeletal stem/progenitor cell (eSSPC). This cluster can generate osteochondral lineage cells.^89^ In summary, the ability of scRNA‐seq to analyse a single cell significantly promotes the exploration of bone development and reveals the complexity of bone cells.

Several additional studies have focused on whole limbs. Because tetrapods are good models for studying the genetic and molecular basis of vertebrate pattern formation, limb development in tetrapods has received extensive research attention.^90^ The genome determines development of the limb.^91^ Feregrino et al used scRNA‐seq to profile transcription patterns in developing chicken autopods.^92^ 17,628 cells were sequenced from three key stages of development in autopods of the chicken, with 1000 genes detected in each cells. Subsequently, 23 cell populations are identified by different transcriptomics patterns. Similarly, Natalie et al study used scRNA‐seq to describe the transcription changes of embryonic hind limb development in mice.^93^ They detected cell heterogeneity, even at the earliest point of development when limb buds could be artificially isolated. The identified cell clusters included the known cell types involved in the development of the hind limb, bone, cartilage and muscle. In addition, to study the limb regeneration mechanisms in salamanders, Gerber et al performed scRNA‐seq in connective tissue cells during bud formation, arm growth and embryonic limb development.^94^ These researchers observed that heterogeneous groups of connective tissue cells converge into a uniform and transient bud‐based progenitor cell state, which is similar to the embryonic limb budding process in later stage. In addition, Qin et al isolated blastema tissues of salamander forelimbs at 3 days, 7 days and 21 days after treated as well as normal forelimbs of salamanders, then conducted scRNA‐seq on 938 extracted cells.^95^ They found a cluster of regenerative cells that is characterized by a significantly higher number of mitochondria than normal limb tissue cells. A novel COL2‐mito subcluster is further defined as COL2^+^ cells, which perform energy metabolite‐related functions, such as responded to oxygen levels and ATP metabolism.

Collectively, these findings demonstrate the ability of scRNA‐seq to isolate populations of developing limb cells at the molecular level. The discoveries of the trajectory of limb development also provide new insights into tissue regeneration in the musculoskeletal system.

### 4.2 Osteoarthritis

OA is a chronic degenerative disease closely related to ageing and progressive joint dysfunction.^96^ The main pathological change of OA is a disorder of articular cartilage homeostasis, accompanied by inflammation and degradation.^97^ Understanding the role and degeneration mechanism of chondrocytes will be helpful to development of an innovative treatment strategy for OA. Ji et al performed scRNA‐seq on 1464 chondrocytes selected from 10 OA patients after knee arthroplasty.^98^ They found that the transcription factors SOX4, TRPS1 and EGR2 are strongly expressed in the early stages of OA and that DNAJC2, GZF1 and ETS2 are strongly expressed in the late stages of OA. Thus, the entire transcription process of degeneration of OA cartilage is described at the single‐cell level, highlighting the potential of cell transcriptomes to diagnose and predict the results of OA cartilage degeneration. Thanks to the unique advantages of SCS in analysing cell heterogeneity, Ji et al found seven potential chondrocyte clusters, including four classical cell clusters and three new clusters. Further research revealed that these chondrocyte clusters played protective or exacerbation role in the OA progression respectively.^99^ Wang et al used scRNA‐seq to identify chondrocyte populations and genetic characteristics of Kashin‐Beck disease, OA and healthy chondrocytes.^100^ Ten cell clusters are annotated by cell markers. A new subcluster, MTCs (mitochondrial chondrocytes), is identified based on the expression of a new set of biomarkers. These findings deepen our knowledge of the functions of OA chondrocytes. More importantly, they also offer new possibilities for treatment strategies of OA.

### 4.3 Intervertebral disc degeneration

Many people suffer from neck pain and lower back pain, which may lead to loss of normal work ability and greatly reduce the quality of life. The main cause of thoracic lumbar pain is intervertebral disc degeneration (IVDD). In recent years, IVDD has become one of the hottest diseases in cell regeneration medicine research.^101^ However, due to the lack of understanding of the molecular characteristic of healthy mature IVD cells, cell regeneration therapies have encountered some obstacles. Li et al chose mature bovine tailbone IVD as the study model because it appeared to be similar in anatomy, histology and biochemistry to human lumbar IVD from healthy young adults aged 15–40 years.^102^, ^103^ The researchers used RISH (RNA in situ hybridization) in bovine IVD to analyse transcriptional target genes in heterogeneous cell populations. They found two new biomarkers for AF (Lam1 and Thy1) and eight new NP biomarkers (Gli1, Gli3, Noto, Scx, Ptprc, Sox2, Zscan10, Loc101904175).^104^ Building on the above result, they used single‐cell resolution FL‐RISH (fluorescent RNA in situ hybridization) to further evaluate and quantify the new biomarkers identified by colour rendering AP‐RISH and z‐scale analysis. Fernandes et al isolated NP and AF cells from healthy human IVD.^105^ They found that FOXM1 and KDM4E transcription factors are key regulators of AF and NP gene networks. Previous research in mouse models had shown that notochord cells contribute to NP. However, retention of these cells in mature humans or bovine NP differs from that in mice or other rodents.^106^, ^107^, ^108^ The difference indicates interspecies heterogeneity of IVD cells. Recently, Gan et al performed scRNA‐seq of 108,108 NP, AF and cartilage endplate (CEP) cells in IVD, providing the most comprehensive systematic single‐cell map of human IVD.^109^ Furthermore, nucleus pulposus progenitor cells (NPPCs) as a specific subset in NP were identified by in vitro functional experiments. These cells have the ability of colony formation and three‐system differentiation. Obviously, the applications of single‐cell technologies have provided a better understanding of the cellular heterogeneity of human IVDs.

### 4.4 Rheumatoid arthritis

Rheumatoid arthritis (RA) is a systemic autoimmune disease mainly involving surrounding joints. RA is characterized by chronic synovial inflammation of the joints, which can lead to erosion and destruction of articular cartilage and surrounding tissues, resulting in joint deformity, stiffness and dysfunction, and finally shortened life span.^110^ Previous studies have demonstrated that the destructive inflammatory environment of the joint synovium results from the action of a variety of cell types, including synovial fibroblasts, macrophages, osteoclasts and vascular endothelial cells.^111^, ^112^ Stephenson et al developed a low‐cost droplet microfluidics control instrument.^113^ They extracted 20,387 cells from synovial tissues of 5 RA patients for single‐cell droplet microfluidics. First, they recognized different lymphocyte populations (T, B and NK cells) in these samples. In the CD4^+^ T‐helper cell population, a unique subgroup of peripheral blood T‐helper cells (TPH) was detected, marked by high levels of MAF, CXCL13 and PDCD1 (PD1). In addition, unbiased cluster analysis reveals three different fibroblast subsets. Two constituent fibroblasts show distinct bifurcation in marker expression (fibroblast 1 and fibroblast 2) and further subdivision of the latter (fibroblast 2a and fibroblast 2b). Fibroblast 1 cells are stained primarily in the synovial lining, while fibroblast 2 cells are marked in the underlying region. Hyaluronic acid synthetase 1 (HAS1) is highly expressed in the lining fibroblasts, suggesting that fibroblasts 1 are responsible for synovium production and turnover. Similarly, Zhang et al performed single‐cell transcriptomics sequencing and flow cytometry analysis of T cells, B cells, monocytes and fibroblasts in synovial tissue from 36 RA patients and 15 OA patients.^114^ The role of specific clusters of cells in RA and chronic inflammation was precisely defined by the integration of cross‐data mode at the single‐cell level. Monocyte samples RNA‐seq data suggested that genes associated with SC‐M1 (IL1B^+^ proinflammatory monocytes) are significantly up‐regulated in leukocyte‐rich RA samples. In contrast, the marker genes associated with SC‐M2 were down‐regulated in OA. In other words, the leukocyte‐rich RA synovium had greater numbers of IL1B^+^ monocytes and IFN‐activated monocytes but lower numbers NUPR1^+^monocytes than the OA synovium. These data suggest that cytokine activation drives the expansion of the unique mononuclear population in the synovial membrane of active RA.

## 5 CHALLENGES AND FUTURE DIRECTIONS

SCS is an emerging technology with great promise, but also with challenges of its application to scientific research. First, the biggest issue is cost, which can easily cost more than $1,000 per sample, including cell capture and library preparation. Second, extraction of single cells is the starting step of SCS. With stroma‐rich bone tissue, it is a difficulty to isolate sufficient amounts and qualities of RNA,^115^ particularly in small animals. Sometimes it is essential to isolate a sufficient number of living cells from the bone marrow or bone tissue, because these living cells accurately represent the cellular diversity of tissues in the body. For instance, multiple displacement amplification (MDA) is the preferred method for genomic DNA amplification, specifically commonly used in low DNA quantities clinical samples.^116^ However, MDA has the defects of amplification bias and unbalanced genome coverage.^117^ In addition, the current data algorithms used in computer bioinformatics still need further development, and new methods are needed to standardize the identification of new cell types.^118^ Professional analysis of the vast amount of sequence data is also a major challenge. Despite these challenges, SCS still has excellent potential. For example, single‐cell technology can be used to perform multi‐layer analyses of the tumour cell genome and transcriptome, which has the potential to revolutionize methods of understanding tumour growth, and this potential exists at every stage of disease development. As we have discussed in this article, SCS has unique superiority to analyse cell heterogeneity in many contexts.^119^ From this perspective, SCS can be applied to musculoskeletal research to identify cell heterogeneity between stem cells populations,^44^ which can help the design of methods to induce specific stem cells to play a role in tissue repair in musculoskeletal diseases.

## 6 CONCLUDING REMARKS

In 2018, ‘development cell by cell’ was named ‘the top one scientific breakthrough of the year’ by *science*.^120^ SCS is the core technology of the researches. Public recognition of this growing and innovative technology shows that SCS has stimulated great changes in scientific research. Over the past decade, investigators have improved or simplified several procedures resulting in drastic reduction in costs. Thanks to the unique advantages of SCS, it is proved to be a technology with broad applications, yielding valuable data on microorganisms, tumorigenesis, and brain or nervous system. As we have seen, this technology has also enabled great advances in the study of heterogeneity of musculoskeletal system (Table 1), and it is believed that the continued application of SCS technologies will achieve more breakthrough in this field. The application of single‐cell technologies in OA has stimulated new potential treatment approaches to this common disease in clinical. Combined with description of developmental trajectory of embryonic limb, SCS technologies can facilitate new cell subtypes discovery and further illuminate the composition of the biological skeleton. A better understanding of bone fracture, ligament tear and post‐traumatic osteoarthritis can lead to come up with innovative treatment strategies to repair bone and joint tissues. In the future, we expect to see more SCS application in limb regeneration, bone metabolism and tumorigenesis in musculoskeletal system.

**TABLE 1:** Cell heterogeneity in the musculoskeletal system researches

Subject	Technology	Method	Result	Reference
Bone development	ScRNA‐seq	CEL‐Seq2	Identification of Ctsk^+^ periosteal stem cell	Debnath et al.^15^
Muscle regeneration	ScRNA‐seq	10X Genomics	Depiction of muscle stem cell homeostatic and regeneration map	Stefania et al.^16^
Limb development	ScRNA‐seq	Fluidigm C1	Identification of SCX^+^ HOXD13^+^ musculoskeletal stem/progenitor cell	Zi et al.^17^
Limb development	ScRNA‐seq	Smart‐seq2	Identification of the Human Skeletal Stem Cell	Chan et al.^18^
Limb regeneration	ScRNA‐seq	10X Genomics	Depiction of chicken autopods developmental transcriptome atlas	Feregrino et al.^19^
Limb development	ScRNA‐seq	10X Genomics	Depiction of embryonic mice hind limb developmental transcriptome atlas	Natalie et al.^20^
Limb regeneration	ScRNA‐seq	10X Genomics	Description of pluripotency in the connective tissue cell of salamander	Gerber et al.^21^
Limb development	ScRNA‐seq	Fluidigm C1	Identification of energy‐metabolite‐related COL2‐mito cells	Qin et al.^22^
Osteoarthritis	ScRNA‐seq	STRT‐Seq	Identification of three new clusters effector chondrocytes	Ji et al.^23^
Osteoarthritis	ScRNA‐seq	10X Genomics	Identification of mitochondrial chondrocytes	Wang et al.^24^
Intervertebral disc degeneration	ScRNA‐seq	Drop‐seq	Description of interspecies heterogeneity of IVD cells	Fernandes et al.^25^
Intervertebral disc degeneration	ScRNA‐seq	10X Genomics	Depiction of systematic single‐cell map of human IVD	Gan et al.^26^
Rheumatoid arthritis	ScRNA‐seq	Drop‐seq (low‐cost droplet microfluidic)	Identification of different lymphocyte populations and two constituent fibroblasts	Stephenson et al.^27^
Rheumatoid arthritis	ScRNA‐seq	CEL‐Seq2	Definition overabundant stromal and immune cell populations in RA	Zhang et al.^28^

## CONFLICT OF INTEREST

The authors declare that they have no competing interests.

## AUTHOR CONTRIBUTIONS

ZY wrote original manuscript. WJ, YC, XK, YB, ZY, YL, WC and HX reviewed and edited. CQ, SL, LF and LC involved in conceptualization, and reviewed and edited. All authors reviewed the final version of manuscript.

## Data Availability

Data sharing is not applicable to this article as no new data were created or analysed in this study.
